# Comparison of Irradiation and *Wolbachia* Based Approaches for Sterile-Male Strategies Targeting *Aedes albopictus*

**DOI:** 10.1371/journal.pone.0146834

**Published:** 2016-01-14

**Authors:** Célestine M. Atyame, Pierrick Labbé, Cyrille Lebon, Mylène Weill, Riccardo Moretti, Francesca Marini, Louis Clément Gouagna, Maurizio Calvitti, Pablo Tortosa

**Affiliations:** 1 CRVOI, Ste Clotilde, Plateforme de Recherche CYROI, Réunion Island, France; 2 Unité Mixte de Recherche «Processus Infectieux en Milieu Insulaire Tropical (UMR PIMIT)», INSERM 1187, CNRS 9192, IRD 249, Université de La Réunion. Plateforme de Recherche CYROI. Ste Clotilde, Réunion Island, France; 3 CNRS, IRD, ISEM - UMR 5554, Université de Montpellier, Montpellier, France; 4 Unité Mixte de Recherche MIVEGEC (IRD 224, CNRS 5290, UM1-UM2), Montpellier, France; 5 ENEA, CR Casaccia, Biotecnologies and Agro-industry Division, Roma, Italy; New Mexico State University, UNITED STATES

## Abstract

The global expansion of *Aedes albopictus* together with the absence of vaccines for most of the arboviruses transmitted by this mosquito has stimulated the development of sterile-male strategies aiming at controlling disease transmission through the suppression of natural vector populations. In this context, two environmentally friendly control strategies, namely the Sterile Insect Technique (SIT) and the *Wolbachia*-based Incompatible Insect Technique (IIT) are currently being developed in several laboratories worldwide. So far however, there is a lack of comparative assessment of these strategies under the same controlled conditions. Here, we compared the mating capacities, *i*.*e*. insemination capacity, sterilization capacity and mating competitiveness of irradiated (35 Gy) and incompatible *Ae*. *albopictus* males at different ages and ratios under laboratory controlled conditions. Our data show that there was no significant difference in insemination capacity of irradiated and incompatible males, both male types showing lower capacities than untreated males at 1 day but recovering full capacity within 5 days following emergence. Regarding mating competitiveness trials, a global observed trend is that incompatible males tend to induce a lower hatching rate than irradiated males in cage controlled confrontations. More specifically, incompatible males were found more competitive than irradiated males in 5:1 ratio regardless of age, while irradiated males were only found more competitive than incompatible males in the 1:1 ratio at 10 days old. Overall, under the tested conditions, IIT seemed to be slightly more effective than SIT. However, considering that a single strategy will likely not be adapted to all environments, our data stimulates the need for comparative assessments of distinct strategies in up-scaled conditions in order to identify the most suitable and safe sterilizing technology to be implemented in a specific environmental setting and to identify the parameters requiring fine tuning in order to reach optimal release conditions.

## Introduction

The Asian tiger mosquito *Aedes albopictus* has emerged as a mosquito species of major medical concern following its global expansion over the past 30–40 years [1–3] and its recent involvement in several arboviral epidemic outbreaks. *Aedes albopictus* is a proven competent vector, in laboratory tests, for over 20 arboviruses including Dengue and Chikungunya viruses [4] and has been shown to be the main vector in a Chikungunya epidemic that hit La Réunion Island in 2005–2006 [5] and Italy in 2007 [6]. This aggressive day-biting mosquito has spread worldwide from its native range in South-East Asia [7, 8] probably mainly disseminating through the international trade of used tires [9]. Its ecological plasticity in different traits including egg diapause, the ability to use natural or urban larval breeding sites [5] and an opportunistic feeding behavior [10, 11] may have favored dispersal and adaptation to newly colonized environments with widely distinct climatic conditions ranging from tropical to temperate [12]. Given the absence of effective vaccines against most of these arboviruses, prevention of epidemics primarily relies on vector control measures. Considerable efforts have been made in order to control wild mosquito populations notably through the use of insecticides. However, their use is increasingly impaired by negative effects on non-targeted organisms and on the environment together with the rapid selection of resistance in insect natural populations [13–15], therefore stimulating the development of innovative control methods.

Among these methods, sterile-male systems aiming at suppressing pest populations using modified males able to introduce sterility in target populations are of particular interest as they are species-specific and environmentally friendly [16–18]. Mating of released sterile males with native wild females may lead to a decrease in the females’ reproductive potential and ultimately to the local elimination or suppression of the pest population if inundative numbers of males are released over a sufficient period of time. The Sterile Insect Technique (SIT), using ionizing radiation to induce random chromosomal rearrangements thus conferring males’ sterility, was the first developed sterile-male system [19]. Several SIT programs have been ever since successfully implemented against a number of agricultural insect pests including the New World screwworm fly or fruit flies, as well as insects of medical importance such as tsetse flies [20, 21] and mosquitoes [22–25]. Several phase 1 studies have demonstrated the potential of SIT in the control of mosquito populations (see [26, 27] for reviews) and field releases of gamma-irradiated *Ae*. *albopictus* males undertaken from 2005 to 2009 in three small Italian towns have shown significant induced sterility in the local mosquito populations [22]. Different techniques have also been developed providing alternative methods to irradiation-based sterilization. These include transgenesis such as the dominant lethal genetic system (RIDL) [28–32] or the use of the endosymbiotic *Wolbachia* [33–38].

*Wolbachia* are maternally inherited Alphaproteobacteria commonly found in arthropods [39], notably in mosquito species of medical importance such as the common house mosquito *Culex pipiens* [40–42] and *Ae*. *albopictus* [36]. In addition, *Wolbachia* infections can be achieved artificially through microinjections as performed in several mosquito species including *Ae*. *albopictus* [43–45], *Aedes aegypti* [46, 47], *Anopheles stephensi* [48] and *Culex tarsalis* [49]. In mosquitoes, *Wolbachia* induce a form of embryonic death called cytoplasmic incompatibility (CI) [50] resulting from sperm-egg incompatibility occurring when *Wolbachia*-infected males mate with uninfected females or females infected with an incompatible *Wolbachia* strain. CI can be either bidirectional when the death of embryos is observed in both reciprocal crosses, or unidirectional when one cross is incompatible while the reciprocal cross is viable. CI can thus be exploited as a source of sterility through a strategy called the Incompatible Insect Technique (IIT) [51], which was first deployed in 1967 in a promising pilot trial carried out in Burma against the filariasis vector *Culex quinquefasciatus* [52]. More recently, encouraging results were also reported under laboratory [33, 35], semi-field [34, 36, 38] and field conditions [37].

In the present study, we compared mating capacities of irradiated (SIT) and CI-inducing (IIT) *Ae*. *albopictus* males in the presence of males and females from La Réunion, a remote oceanic island lying 700 km East of Madagascar that has experienced a major Chikungunya epidemics in 2005–2006 [53]. The magnitude of the epidemics, which infected a third of the Island population, led health authorities to dramatically strengthen vector control measures mainly through the use of larvicides (temephos, then *Bacillus thuringiensis var israelensis* or Bti) and adulticides (fenitrothion, then deltamethrine). Following a restriction on organophosphates, the vector control unit was left with pyrethroids and Bti as the only available insecticides. This challenging situation has stimulated research programs aiming at assessing the feasibility of environmental-friendly strategies such as SIT, developed in an area-wide integrated pest management (AW-IPM) program targeting *Ae*. *albopictus* [25, 54, 55], and IIT [35, 38]. As both SIT and IIT are currently being developed in La Réunion, we took advantage of this unique opportunity to compare the competitiveness of irradiated and incompatible *Ae*. *albopictus* males with the goal of pinpointing the potential strength and drawbacks of each strategy.

One of the key parameters needed for the evaluation of a sterile-male system is the actual mating competitiveness of released males since these must compete with wild males in seeking and inseminating wild females. Male mating competitiveness is dependent on several parameters such as survival rate, mating capacity and sterilizing properties of the inseminated sperm, all of which can be affected by irradiation treatment or by *Wolbachia* infections. Irradiation was shown to affect competitiveness of *Ae*. *albopictus* males when performed at a dose inducing nearly full sterility (40 Gy) [56, 57], while the artificial infection with *w*Pip *Wolbachia* was not found to decrease male competitiveness even if inducing full sterility [36]. Here, we examined the (i) insemination capacity, (ii) sterilizing capacity and (iii) mating competitiveness of irradiated and incompatible *Ae*. *albopictus* males under laboratory-controlled conditions. Results presented herein provide important insights on the relative effectiveness of SIT and IIT for the control of *Ae*. *albopictus* natural populations, and potential drawbacks and associated improvements of each technique are discussed.

## Materials and Methods

### Mosquito collections

Two *Ae*. *albopictus* lines were used in the experiments: the wild type LR line naturally co-infected with two *Wolbachia* strains (*w*AlbA and *w*AlbB) and established from approximately 1000 eggs sampled in three localities of La Réunion Island in 2012 (Saint Denis, Sainte Suzanne and Saint Benoît); and the AR*w*P_IT_ line previously constructed using embryonic microinjections of eggs’ cytoplasm (including *Wolbachia w*Pip infections) from *Cx*. *pipiens* [43]. As the AR*w*P_IT_ line was generated with *Ae*. *albopictus* mosquitoes from Italy and in order to limit the influence of the nuclear genome of Italian origin, the cytoplasm (including *w*Pip infections) of the AR*w*P_IT_ line was introduced into the nuclear background of the LR line through 4 consecutive backcrosses (100 virgin AR*w*P_IT_ females crossed with 100 LR 2 weeks aged males) expected to restore ~ 90% of LR nuclear genes. In fact, AR*w*P_IT_ females have been reported to be partially compatible (about 20% fertile) when mated with wild-type males aged more than two weeks, due to a mean reduction in *w*AlbA *Wolbachia* titer [58]. Thus, the AR*w*P_LR_ line carrying most of the LR nuclear genome together with a *w*Pip infection was obtained and further used in the experiments. *Wolbachia* infections were controlled in the LR and AR*w*P_LR_ lines through a PCR genotyping procedure using the ankyrin domains *ank2* gene, which is specific to *w*Pip infections [42, 59]. Therefore, no PCR amplification was observed in samples from the LR line whilst a PCR amplified fragment of 511 bp was observed using samples from the AR*w*P_LR_ line.

Mosquitoes were reared in the laboratory under standard conditions at 27 ± 2°C and 75 ± 2% relative humidity (RH) and an LD 12:12 h photoperiod. Larvae were fed *ad libitum* with a mixture of rabbit and fish-food. Adults were maintained in cages (30×30×30 cm) and fed *ad libitum* with 10% sucrose solution. Females were fed on sheep blood and collected eggs were stored at room temperature. Experiments were performed with G30 and G4 generations for LR and AR*w*P_LR_ lines, respectively.

### Ethics statement

No specific permissions are required for the field activities as they do not involve endangered or protected species and field sites where eggs were sampled are not privately-owned. In addition, blood meals were carried out using artificial systems containing sheep blood that was purchased from a qualified supplier (Prolab, Saint-Pierre, Réunion Island), thus avoiding the need for any vertebrate as blood-feeding source.

### Irradiation procedure

Batches of 20 to 24 hours old male pupae from the LR line were maintained in 4 cm wide cups filled with dechlorinated water and further exposed to gamma rays emitted by a cesium-137 irradiator (IBL 437, Cis Bio International, Germany). The chosen irradiation dose was 35 Gray (Gy) delivered at 2.35 Gy/min, consistent with previous investigations [24, 25]. This sterilizing condition doesn’t induce full sterility but was shown to have limited adverse effects on *Ae*. *albopictus* males competitiveness [24, 25]. Hence, all data presented herein were produced using specimens from the LR line irradiated with 35 Gy and thereafter named LRi.

#### Insemination and sterilizing capacities

Following emergence of adults, different batches of twenty virgin LR females aged 3–5 days were mixed with 20 LR, LRi or AR*w*P_LR_ males in 30×30×30 cm cages. Tests were performed using either 1, 5 or 10 day-old virgin males, with the day of emergence being considered as day 0. Three replicates were performed for each type of cross thus requiring a total of 27 cages: 9 cages with 1 day-old males, 9 cages with 5 day-old males and 9 cages with 10 day-old males. Males and females were allowed to mate for 24 h. Males were then removed from cages using a mouth aspirator and wing size was measured. Females were blood fed two days after the removal of males and laid eggs were collected, counted, dried for four days under laboratory conditions and then allowed to hatch. A second blood meal was given to females 10 days following the first one and eggs were subsequently collected and treated under the same conditions as for the first batch.

Male size was assessed using the left wing of individuals dissected under a binocular microscope (Leica MZ 6). The wing length was measured by considering the distance from the distal edge of the alula to the end of the radius vein (excluding fringe scales). A digital image of the wing was captured using a ScopeTek DCM310 camera mounted on the binocular microscope and the measures were performed using a custom-written MATLAB-based user interface. Scales were determined capturing an image of a micrometer.

To assess male insemination capacity, surviving females were collected after oviposition and dissected on a slide under a binocular microscope (Leica MZ 6) with 80X magnification. Spermathecal capsules were isolated, torn gently with micropins and checked for the presence of spermatozoa. Two parameters were then recorded: (a) the number of inseminated females (*i*.*e*. found with at least one filled spermathecal capsule), and (b) the number of females with 0, 1, 2 or 3 filled spermathecal capsules amongst inseminated females.

Sterilizing capacity of males was examined by comparing egg-hatching rates in sterile crosses (*i*.*e*. between ♂LRi×♀LR or ♂AR*w*P_LR_×♀LR) to that of the fertile control cross (*i*.*e*. between ♂LR×♀LR).

#### Mating competitiveness

Confrontations were carried out by mixing 20 virgin LR females with different ratios of sterile to LR males’ in cages of 30×30×30 cm. Two ratios were tested: 1:1 (20♂LRi+20♂LR or 20♂AR*w*P_LR_+20♂LR) and 5:1 (100♂LRi+20♂LR or 100♂AR*w*P_LR_+20♂LR). All females were 3 to 5 day-old, whilst virgin males of 1, 5 and 10 day-old were used in the different confrontations. Three replicates were performed for each type of confrontation, thus requiring a total of 36 cages: 18 trials for the 1:1 ratio (6 with 1 day-old males, 6 with 5 day-old males and 6 with 10 day-old males) and 18 trials for the 5:1 ratio (6 with 1 day-old males, 6 with 5 day-old males and 6 with 10 day-old males). Males were first released in cages followed by females and mosquitoes were allowed to mate for 24 h. Thereafter, males were removed from cages using a mouth aspirator and females were blood fed in the laboratory two days after the removal of males. Eggs were collected, counted, dried for 4 days under laboratory conditions and then allowed to hatch in order to measure hatching rates. A second blood meal was given to females 10 days after the first one to enable further oviposition. This second batch of eggs was treated in the same way as the first one.

### Data analysis

Male wing size was analyzed using the GLM Size = Type + *ε*, where Size is the quantitative response variable and Type a three-level factor corresponding to LR, LRi and AR*w*P_LR_ males. *ε* represents the error term, following a binomial distribution. Normality of the model residuals was tested using a Shapiro-Wilk test (Shapiro).

Insemination capacity was analyzed using the GLM Fi = Type + Age + Type:Age + *ε*, where Fi, the response variable, corresponds to the proportion of inseminated females, Type a three-level factor corresponding to LR, LRi and AR*w*P_LR_ males, and Age a three-level factor corresponding to the number of days between emergence and the assay (1, 5 or 10 days). *ε* represents the error term, following a binomial distribution. The ":" represents the interaction between the two factors. The distributions of the number of filled spermathecae (among inseminated females) were compared between the different types of males at each age using Fisher exact test.

Hatching rate (egg fertility) was calculated for each individual cage by dividing the number of hatched eggs by the total number of laid eggs. It was analyzed using the GLM Hr = Type + Age + Type:Age + *ε*, where Hr, the response variable, corresponds to the proportion of hatching eggs, Type a three-level factor corresponding to LR, LRi and AR*w*P_LR_ males, Age a three-level factor corresponding to the number of days between emergence and the assay (1, 5 or 10 days). *ε* represents the error term, following a binomial distribution. The ":" represents the interaction between the two factors.

The competitive index, ‘C’, defined by Fried [60] was calculated for each type of confrontation using hatch rates from the fertile control (Hn, *i*.*e*. between ♂LR×♀LR), sterile control (Hs, *i*.*e*. between ♂LRi×♀LR or ♂AR*w*P_LR_×♀LR) and the confrontation cages (Ho, *i*.*e*. ♂LRi×♂LR×♀LR or ♂AR*w*P_LR_×♂LR×♀LR) as follows: C = N/S × Hn-Ho/Ho-Hs, where N is the number of wild type LR males and S is the number of sterile males (irradiated LRi males or incompatible AR*w*P_LR_ males). Values around 1 indicate equivalent mating performance between sterile and wild type males. To evaluate the effects of sterile male releases on the cage confrontations’ resulting fertility, the induced sterility (IS) was calculated as 100% minus the residual fertility value, which was obtained by dividing the observed hatch rate (Ho) by the control hatch rate (Hn).

All computations were performed using the R free software (v.3.1.1, http://www.r-project.org). GLM were simplified as follows: significance of the different terms was tested starting from the higher-order terms using likelihood ratio test (LRT). Non-significant terms (*P*> 0.05) were removed [61]. Factor levels of qualitative variables that were not significantly different were grouped (LRT, [61]).

## Results

### Irradiated and incompatible males have lower insemination capacities at emergence

The insemination rates of wild type LR females caged for 24 h with wild type LR males, irradiated LRi males or incompatible AR*w*P_LR_ males were examined by assessing the percentage of inseminated females and the percentage of females with 0, 1, 2 or 3 filled spermathecal capsules.

There was no interaction effect between males’ age and type on the percentage of inseminated females (Male:Type, LRT, *X* = 6.3, *Δdf* = 4, *P* = 0.35). However, LR males were more successful in inseminating females (LRT, *X* = 21.1, *Δdf* = 2, *P*< 0.001, no significant difference between LRi and AR*w*P_LR_ males, LRT, *X* = 2.02, *Δdf* = 1, *P* = 0.15), but only at day 1 (LRT, *X* = 117.7, *Δdf* = 2, *P*< 0.001, no significant difference between day 5 and 10, LRT, *X* = 0.2, *Δdf* = 1, *P* = 0.64; Table 1). We then analyzed the number of filled spermathecae in inseminated females at day 1. It appeared that AR*w*P_LR_ males filled significantly less spermathecae that the other males (Fisher exact test, *P*< 0.001; Table 1), since they mostly filled only one spermathecae while LR and LRi males usually filled two (no significant difference between LR and LRi distributions, Fisher exact test, *P* = 0.44).

**Table 1:** Insemination capacity of irradiated and incompatible males caged with females for 24 h.

Male	Age at release	Number of females	Percentage of inseminated females (N)	Percentage of filled spermathecae (N)			
				0	1	2	3
LR	1 day-old	57	74% (42)	26% (15)	12% (7)	58% (33)	4% (2)
LRi	1 day-old	54	46% (25)	54% (29)	4% (2)	43% (23)	0% (0)
AR*w*P_LR_	1 day-old	56	34% (19)	66% (36)	21% (12)	13% (7)	0% (0)
LR	5 day-old	47	100% (47)	0% (0)	0% (0)	89% (42)	11% (5)
LRi	5 day-old	57	91% (52)	9% (5)	2% (1)	86% (49)	4% (2)
AR*w*P_LR_	5 day-old	54	91% (49)	9% (5)	9% (5)	80% (43)	2% (1)
LR	10 day-old	56	93% (52)	7% (4)	0% (0)	88% (49)	5% (3)
LRi	10 day-old	56	95% (53)	5% (3)	4% (2)	89% (50)	2% (1)
AR*w*P_LR_	10 day-old	52	90% (47)	10% (5)	6% (3)	83% (43)	2% (1)

Three types of males (*i*.*e*. LR = wild type, LRi = wild type irradiated and AR*w*P_LR_ = incompatible) aged of 1, 5 or 10 days were compared.

### Incompatible and irradiated males display distinct sterilizing capacities over their lifespan

We measured the strength of induced sterility through crosses involving LR females and LRi or AR*w*P_LR_ males. The fertility of LR females in control crosses with LR males of 1, 5 and 10 day-old was 77.0%, 56.1% and 83.3%, respectively (Table 2). Irradiated males showed a residual fertility when crossed with LR females; the hatching rates were 6.1%, 4.6% and 8.1% with males aged of 1, 5 and 10 days, respectively (Table 2; significant age effect: LRT, *X* = 30.8, *Δdf* = 2, *P*< 0.001). These hatching rates were significantly higher than those measured in crosses with AR*w*P_LR_ males (LRT, *X* = 665.4, *Δdf* = 1, *P*< 0.001) where total sterility (*i*.*e*. hatching rate = 0%) was indeed recorded regardless males’ age (Table 2). In addition, there was no significant variation in the hatching rates between the first and second batch of eggs (*i*.*e* after a second blood meal; LRT, *X* = 1.9, *Δdf* = 1, *P* = 0.17).

**Table 2:** Sterilizing capacity of irradiated and incompatible males.

Crosses		1 day-old		5 day-old		10 day-old	
(males × females)		N. eggs	Hatching rate (95% CI)	N. eggs	Hatching rate (95% CI)	N. eggs	Hatching rate 95% CI
Sterile	LRi × LR	1772	0.061 (0.051–0.073)	2600	0.046 (0.038–0.055)	3243	0.081 (0.072–0.091)
	AR*w*P_LR_ × LR	1271	0 (-)	3799	0 (-)	2693	0 (-)
Fertile	LR × LR	2365	0.770 (0.752–0.787)	2972	0.561 (0.543–0.579)	2606	0.833 (0.818–0.847)

Males (N = 20) were allowed to mate with females (N = 20) for 24 h. Three replications were performed for each type of cross and two series of eggs were collected. LR = wild type, LRi = wild type irradiated, AR*w*P_LR_ = incompatible. The hatching rates are indicated with their 95% confidence intervals (95% CI). Note that the two batches were pooled as there was no significant difference (see text).

### Variability in mating competitiveness of irradiated and incompatible males according to age and ratios

Experiments were carried out to examine the hatching rate and the competitiveness index (C) of LRi and AR*w*P_LR_ males in competition with LR males for inseminating LR females. Males were released at 1, 5 and 10-day old in a 1:1 or a 5:1 ratio.

Hatching rate analysis revealed strong interactions between males’ age, males type and the ratios of sterilizing males implemented during the trial (Type:Age:Ratio interaction, LRT, *X* = 550.9, *Δdf* = 2, *P*< 0.001). Interactions between males’ type and age remained significant even when analyzing the ratios independently (Type:Age interaction, LRT, *X* = 465.1, *Δdf* = 2, *P*< 0.001 for the 1:1 ratio; *X* = 202.2, *Δdf* = 2, *P*< 0.001 for the 5:1 ratio). Nevertheless, a global observed trend is that AR*w*P_LR_ males tend to induce a lower hatching rate than LRi males (Table 3).

**Table 3:** Mating competitiveness of irradiated (LRi) and incompatible (AR*w*P_LR_) *Ae*. *albopictus* males in cages containing either a 1:1 or a 5:1 ratio with respect to wild-type males (LR).

Ratio	Male age	Crosses	Number of eggs	Number of hatched eggs	Hatching rate (95% CI)	Fried index C (95% CI)	Induced sterility (IS)
1:1	1-day-old	♂LRi×♂LR×♀LR	2329	1846	0.793 (0.775–0.809)	-0.031 (0.073–0.078)	-0.029
		♂AR*w*P_LR_×♂LR×♀LR	1907	960	0.503 (0.481–0.526)	0.530 (0.154–0.181)	0.346
	5-day-old	♂LRi×♂LR×♀LR	2091	687	0.327 (0.309–0.349)	0.824 (0.299–0.397)	0.415
		♂AR*w*P_LR_×♂LR×♀LR	3644	1188	0.326 (0.311–0.341)	0.723 (0.251–0.311)	0.419
	10-day-old	♂LRi×♂LR×♀LR	3833	1456	0.380 (0.365–0.395)	1.516 (0.369–0.514)	0.544
		♂AR*w*P_LR_×♂LR×♀LR	2028	1033	0.509 (0.488–0.531)	0.635 (0.156–0.185)	0.388
5:1	1-day-old	♂LRi×♂LR×♀LR	2444	1269	0.519 (0.499–0.539)	0.109 (0.034–0.041)	0.326
		♂AR*w*P_LR_×♂LR×♀LR	2664	989	0.371 (0.353–0.390)	0.215 (0.051–0.063)	0.518
	5-day-old	♂LRi×♂LR×♀LR	3173	592	0.187 (0.173–0.201)	0.533 (0.173–0.303)	0.668
		♂AR*w*P_LR_×♂LR×♀LR	4048	546	0.135 (0.125–0.146)	0.633 (0.179–0.268)	0.760
	10-day-old	♂LRi×♂LR×♀LR	2413	1218	0.505 (0.485–0.525)	0.155 (0.040–0.050)	0.394
		♂AR*w*P_LR_×♂LR×♀LR	2564	440	0.172 (0.158–0.187)	0.770 (0.177–0.255)	0.794

The hatching rates and the values of the Fried index’s (C) are indicated with their 95% confidence intervals (95% CI).

In the 1:1 ratio, the competitiveness index (C) for LRi males were: -0.031, 0.824 and 1.516; and the induced sterility (IS) was: -2.9%, 41.5% and 54.4% for 1, 5 and 10 day-old males, respectively (Table 3). The estimated C values for AR*w*P_LR_ males were: 0.530, 0.723 and 0.635; and the IS values were: 34.6%, 41.9% and 38.8% for 1, 5 and 10 day-old males, respectively (Table 3). The comparison of mating competitiveness of LRi and AR*w*P_LR_ males in the 1:1 ratio showed a better competitiveness of the former at 10 days and of the latter at 1 day, respectively; while the difference between the two types of male was not significant at 5 days. When the 5:1 ratio was implemented, the estimated C values were: 0.109, 0.533 and 0.155; and IS values were: 32.6%, 66.8% and 39.4% for 1, 5 and 10 day-old LRi males, respectively (Table 3). As for AR*w*P_LR_ males, the estimated C values were: 0.215, 0.633 and 0.770; and the IS values were 51.8%, 76% and 79.4% for 1, 5 and 10 day-old males, respectively (Table 3). Thus, the comparison of mating competitiveness of LRi and AR*w*P_LR_ males in the 5:1 ratio showed a better competitiveness of AR*w*P_LR_ males regardless of age.

**Fig 1:**
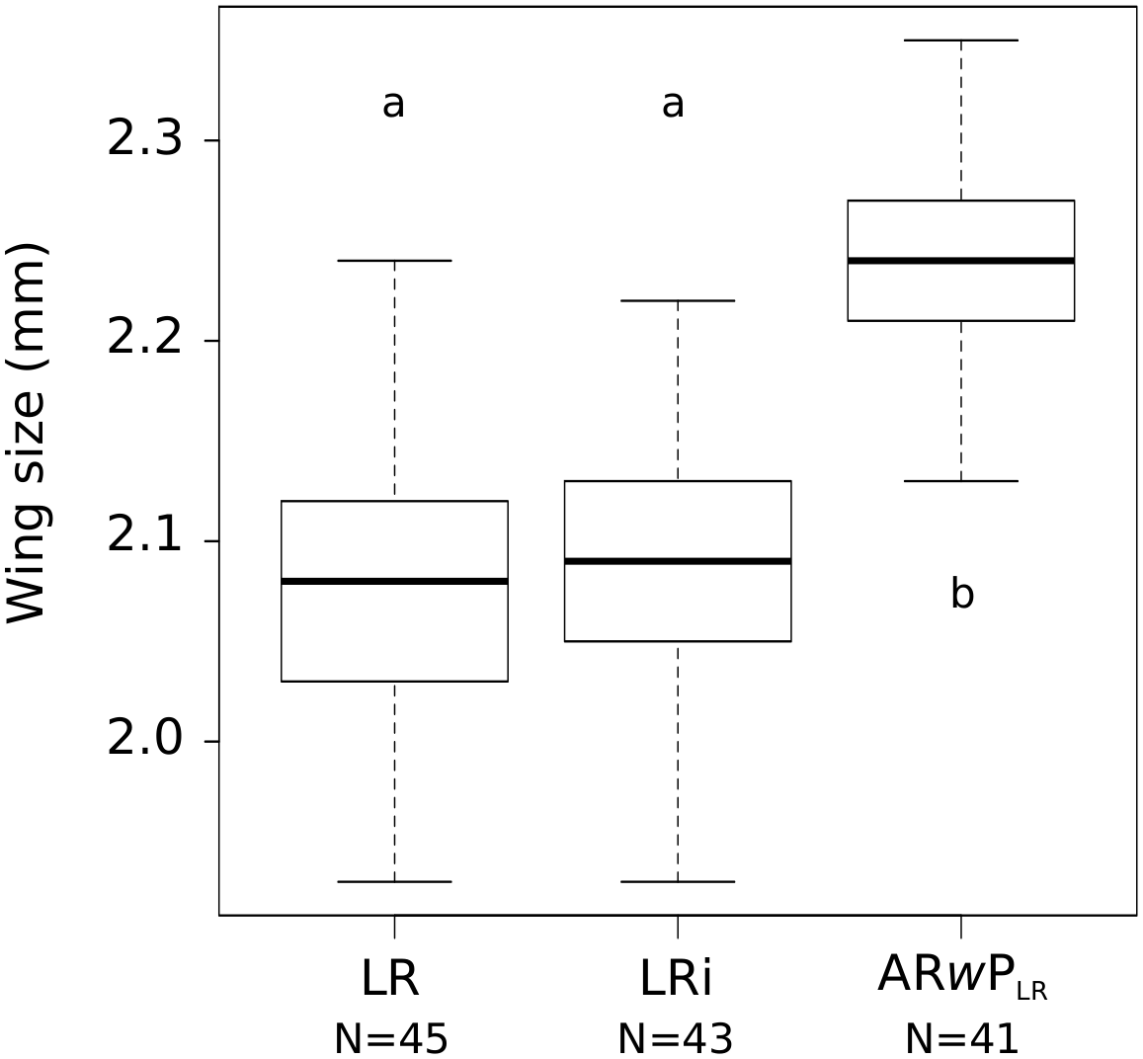
Wing size of males. LR = wild type males, LRi = wild type irradiated males and AR*w*P_LR_ = incompatible males. N = number of mosquitoes measured. a and b represent statistical group (LRT).

Finally, as male mating competitiveness could be affected by size we measured male wings’ size as a proxy of adult size for all male types. As shown on Fig 1, irradiation of LR pupae did not affect wing size of emerging adults as there was no significant difference between untreated males and their irradiated counterparts (LRT, *F* = 2.66, *P* = 0.11). However, AR*w*P_LR_ males were significantly larger than the other males (LRT, *F* = 101.4, *P*< 0.001, Fig 1).

## Discussion

Several environmental friendly vector control strategies willing at reducing disease transmission through the control of vector populations are currently under development. Among these, sterile-male release strategies are being implemented at pilot scales in several countries [18, 27]. Although laborious, as they require the consistent mass production of sterile males, sterile-male strategies provide the benefits of strong species specificity while implementation may be stopped at any time. In mosquitoes, males’ sterility can be achieved through irradiation, *Wolbachia*-based CI or transgenesis. Yet no attempt has been made to directly compare these approaches in order to provide insight into their relative efficacy in the same experimental framework. To address this gap, we conducted a series of laboratory experiments aiming at comparing mating capacities of irradiated (LRi) and incompatible (AR*w*P_LR_) males.

Crosses between AR*w*P_LR_ males and wild type females showed total sterility regardless of male age and number of blood meals offered to the females; while 5–8% of hatched eggs were observed in crosses involving LRi males, in keeping with previous investigations using the irradiation dose of 35 Gy [24, 25]. Several studies have addressed the effect of increasing irradiation on induced sterility and other life history traits. An increased dose will lead to full sterility of the treated males but may also affect life expectancy and mating competitiveness and, as a result, the capacity of irradiated males to reduce egg hatch when confronted to wild males [56, 57]. Indeed, Bellini et al. [56] showed that irradiation doses of 30 and 40 Gy were both considered as the best compromise since the reduction in male sterility was overcompensated by the increased competitiveness of partially fertile males. So, it is in the interest of SIT programs to choose radiation doses representing the best compromise between sterilization and mating competitiveness, as in the case reported for the fly *Anastrepha obliqua* [62].

The insemination capacities of LRi and AR*w*P_LR_ males were similar, both male types showing lower capacities at 1 day as compared to wild type males, but recovering full capacities 5 days after emergence. Hatching rate measured in cages confrontations revealed strong interactions between the types and ages of males and the ratios of sterilizing males used in the trial. In the 1:1 ratio, hatching rates from cages with LRi males at 5 and 10 days were lower than that recorded in cages with AR*w*P_LR_ males; whereas in the 5:1 ratio, all hatching observed in cages with AR*w*P_LR_ males were lower than that observed in cages where LRi males were introduced. However, generally, AR*w*P_LR_ males tended to induce a lower hatching rate (and thus superior sterility, Table 3) than LRi males under the tested conditions. The difference in mating competitiveness of LRi and AR*w*P_LR_ males according to the ratio could be explained by the density of mosquitoes within experimental cages. Indeed, all confrontations were performed in identical cages of 30×30×30 cm, and 40 males (20 LRi or 20 AR*w*P_LR_ + 20 LR) were released in cages with the 1:1 ratio whereas 120 males (100 LRi or 100 AR*w*P_LR_ + 20 LR) were released in cages with the 5:1 ratio. The effect of mosquito density on hatching has been previously described by Madakacherry et al. [24] who observed higher hatched eggs in small laboratory cages compared to large laboratory cages containing the same number of mosquitoes. So, in competition trials, mating and inseminating capacities may depend on insect densities. The C values for LRi and AR*w*P_LR_ males were lower than 1 in general, suggesting a lower mating competitiveness of both male types as compared to wild-type males. A lower competitiveness of AR*w*P_LR_ males may result from insufficient number of backcrosses. Indeed, although the cytoplasm of AR*w*P_IT_ was introgressed into a LR nuclear background through four successive backcrosses, we cannot exclude a nuclear effect of the remaining (theoretically 10%) AR*w*P_IT_ nuclear genome that may somehow reduce mating capacities of AR*w*P_LR_ males with LR females. Thus, in future investigations, a greater number of backcrosses between transinfected and wild type mosquitoes will be performed in order to avoid side effect of nuclear genes on mating capacities of incompatible males. Surprisingly, the C value of LRi males at 1 day was negative due to the higher hatching rate in competition trials as compared to fertile control (see Tables 2 and 3). While the reason for this higher hatch is not clear, human error during egg maturation and counting or problems with the blood cannot be ruled out.

We found an effect of male age in mating competitiveness. Generally, the fertility of LR females was significantly lower in cage confrontations where 5 and 10 day-old LRi and AR*w*P_LR_ males were released, as compared to cages with 1 day-old males. Several factors including magnitude and persistence of induced sterility as well as delayed sexual maturation may account for this transitory mating competitiveness. We measured both strength and persistence of induced sterility in LRi and AR*w*P_LR_ males. We observed a residual fertility in matings with LRi males in agreement with previous investigations showing the existence of residual fertility in crosses between *Ae*. *albopictus* females and 35 Gy irradiated males [24, 25]. Although there was a significant difference in hatching rates between age classes, this difference was not consistent with a mating competitiveness increasing with age. As for *Wolbachia*, it has been shown that *Wolbachia*-induced sterility can change with male age as observed in some arthropod species [63–66] including *Ae*. *albopictus* [58, 67–69]. Crosses between AR*w*P_LR_ males and wild type females led to full sterility, thus confirming that CI properties of AR*w*P_IT_ males, previously shown to induce total embryonic mortality in crosses with wild type females [36, 43, 69], was not altered by the nuclear background of the LR line. Importantly, the levels of sterility remained constant in 1, 5 and 10 day-old males, hence allowing rejecting the hypothesis of any change of *Wolbachia*-induced sterility with males’ age. Lastly, we addressed the effect of male age on insemination capacity. Irradiated LRi and AR*w*P_LR_ were less successful in inseminating females at 1 day as compared to LR, and no significant difference was noted between the three male types at days 5 and 10. To become sexually mature, males must complete several physical changes including complete rigidity of antennal fibrillar hairs and a 180° rotation of the terminalia part of the genitalia after emergence (see [70] for review). The time required to complete terminalia rotation varies between mosquito species. For instance, 18 to 24 h was reported for *A*. *aegypti* [71] and 11 to 25 h for *A*. *albopictus* [72]. Oliva et al. [72] showed that genitalia rotation was slightly delayed in irradiated males although they did not observe statistical difference in insemination rates according to age. However, comparing our data to this previous study is not straightforward since we allowed females to mate for 24 h while Oliva and co-workers allowed females to mate for 48 h, which must have significantly increased the insemination rate as previously shown for *Anopheles coluzzii* [23]. As far as *Wolbachia* is concerned, there is no study describing any delay in sexual maturation for artificially infected *Ae*. *albopictus* lines, so future investigations comparing sexual maturation of irradiated and incompatible males should investigate this point of interest. Although we cannot clearly demonstrate the correlation between insemination capacity and mating competitiveness, this factor should definitely be considered for the implementation of SIT or IIT in the field. The effect of male life history and mating behavior has been investigated theoretically for sterile-male release programs [73]. The results suggest that if male mating capacity increases over the first week of life and if released males suffer a mortality cost, older males should be released due to their increased mating capacity [73]. So, in the context of an operational implementation of SIT and/or IIT, irradiated or incompatible males should be released in the field at their maximum competitiveness, while devices allowing the emergence of sterile males *in natura* should be avoided.

Although SIT and IIT are vector control techniques based on a common strategy, *i*.*e*. the inundative release of sterilizing males, each method has its pros and cons. Due to residual fertility observed with irradiated (35 Gy) males, an operational implementation of SIT using this radiation dose may require a greater number of irradiated males to be released than IIT. On the other hand, although both techniques require selective release of males only, IIT is extremely sensitive to accidental release of females, especially when repeated releases have suppressed local mosquito populations making any female release favorable to population replacement. Nevertheless, bidirectional CI occurring between incompatible and wild mosquitoes (including mosquitoes immigrating into the treated area) is expected to significantly lower the population replacement risk. Overall, our data reveal that incompatible males were slightly superior to irradiated males under the tested conditions. However, this study may highlight a possible complementarity of both techniques. Indeed, application of low dose of irradiation with the aim of sterilizing females without affecting the quality of the released males could lower the risk of accidental releases of incompatible females [74–76]. We propose an alternative approach: IIT implementation requires a surveillance of *Wolbachia* infection in treated populations all over releases, and the appearance of any sign of population replacement may be overcome by the release of irradiated males. Indeed, our data suggest that incompatible males are more competitive in the presence of high densities of mosquitoes and irradiated males in low densities. Given that the accidental release of incompatible females is most risky in the presence of low densities of mosquitoes, SIT and IIT may be used successively based on an evaluation of the density of mosquito populations in targeted sites. Of course, the availability of an irradiator together with the cost of such combination need to be taken into consideration. Altogether, our results call for additional experiments comparing life history traits and mating competitiveness of irradiated and incompatible males under semi-field conditions since discrepancies between laboratory and field cage experiments have been previously reported [32].

### Conclusion

In this study, we compared mating capacities of irradiated and incompatible males. Both males showed a better competitiveness with ageing, highlighting the need for accurate analyses of male life history traits and mating behavior for sterile-male release strategies in order to increase the effectiveness of SIT and IIT control programs. Our data suggest that both techniques would benefit from a release of males a few days after emergence. Altogether, the analysis of mating competitiveness revealed that incompatible males were slightly superior to irradiated males. However, since the possible fate of AR*w*P_LR_ females, eventually co-released in the field, was still not ascertained by a rigorous risk assessment protocol, our comparative data highlight that both techniques are likely complementary and may be indeed implemented alternatively or in combination within a same treated area. Comprehensive investigations in semi-field conditions will allow selecting for the most relevant strategy at a specific site and fine-tuning the releasing conditions with the aim of maximizing the success of these appealing environmental-friendly strategies.
